# Gleanings from Our Exchanges

**Published:** 1872-08-01

**Authors:** 


### Gleanings from Our Exchanges.

### THE USE OF PEPSINE IN THE DIARRHŒA OF INFANTS.

We make the following extract from a pamphlet on this subject by James S. Hawley, M.D., of Brooklyn, N. Y.:

We have now briefly noticed, in outline, the first two conditions of treatment, viz.: the removal of external causes of irritation, and the allaying of the morbid excitement which has sprung from their agency; and it may be asked if the natural functions will not now resume their offices, and the health of the patient be restored. Doubtless such would be the case did not the system labor under the combined effects of the transition state of dentition, and the impairment of strength due to the morbid causes above enumerated, and for which the correctives have been proposed. But the circle of remedies is not yet complete. The keystone of the arch is wanting, and, if left thus incomplete, will tumble back into the disorder and confusion from which it has been raised. The *ingesta* themselves become, for want of digestive and assimilative power, irritants to the sensitive and debilitated organs. Instead of affording nutriment to fortify the system against the dangers of the crisis through which it is passing, the food going through the intestinal canal in an undigested form, becomes, itself, an irritant, and adds another morbid cause to those already existing. This is not all; the food does not always remain a simple, foreign substance, inducing irritation, but undergoes putrifactive decomposition, adding new and more active sources of disease.

Here, then, is the gap in which we are to stand. And what are the weapons we are to use? Tonics and stimulants, as indispensable as they often are, here become awkward and doubtful. They are indirect and secondary —whips to stimulate lagging energies, and not power to perform the labor or lift the burden. Here the happy thought of Corvisart comes to our relief. The very function which is crippled, we can replace; the very strength which is exhausted, we can supply. By the administration of pepsine, we at once convert the ingesta into nutriment. They not only cease to be irritants to the digestive organs, but are absorbed into the circulation, and become sources of power instead of weakness.

Now we have fulfilled all the indications. First, to remove all sources of irritation from the quantity or quality of the ingesta, or change of temperature. Secondly, allaying irritation by sedatives. Thirdly, artificial digestion by the administration of pepsine. This simple but effective treatment is not new, but has more than once been presented to the profession for its approval.

In support of its efficacy, especially that portion which relates to artificial digestion, and which the paper is particularly intended to illustrate, a number of cases are brought forward, of which the two following are samples:

Case 8. July 28th. D. N——, an infant two weeks old, said to have been born in a plump and healthy condition. Its present state is one diametrically opposite. Its face is thin and skinny, exhibiting painfully the bony outline. It has thin, muddy, but not frequent alvine discharges, and vomits whatever it swallows, even to half a teaspoonful of its mother's milk. It lies stupid, with its eyes closed, and refuses the breast. It also has an intense muguet. In this extremity I ordered three grains of pepsine to be given every three hours, and half a teaspoonful of the mother's milk to be administered with great frequency. The following morning I found the mother, through utter hopelessness, had greatly neglected my directions. It was only through much persuasion, and the co-operation of a friendly neighbor, that she was induced to pursue the treatment. During that day the vomiting ceased, and on the following day the child took the breast and retained and digested its nourishment. From this day it steadily improved in condition, and its diarrhœa and muguet disappeared.

On the 8th inst., one week after my last visit, I was called to see its mother, and could hardly have recognized the infant which so lately had seemed in the last stage of inanition. Its face had acquired a comparative fullness, its color was restored, it nursed well and freely, and seemed as likely to live and thrive as any infant. This child was simply starving to death. What led to its condition of inanition I could not satisfactorily learn, but its state seemed most hopeless. This case illustrates, in a remarkable manner, how little assistance will restore the digestive faculty to its normal activity, and enable it to perform its functions unassisted.

Case 9. Christian Schwindt, aged 2½ years. The child has been suffering about six months from diarrhœa, and has become extremely emaciated and pallid. His food is passed undigested soon after ingestion. He has been under treatment during the greater part of his illness, without success, and one of his medical attendants had pronounced the case incurable.

I ordered 15 grs. of the Am. Pepsine every time food was taken. The stools immediately began to show less undigested food, and the diarrhœa to abate. In four or five days the diarrhœa ceased, and flesh began to return to the emaciated frame.

At the time of making this note, one month after the administration of the pepsine, he is in perfect health.

Another great advantage arising from the use of pepsine in this disease, has been rendered apparent by a careful comparison of cases treated by the most approved method in vogue some eight years ago. The medical gamut was then sedatives, opiates, astringents, tonics. It yielded good results, but the cases were a long time in getting well. There came a period when the pernitrate of iron and the salts of quinia seemed almost powerless. This might be the third week or the third month in the disease. There was no particular irritation of the stomach, but unless the astringent was given with great regularity and in augmented doses, the discharges still continued. In these protracted cases the gastro-intestinal system seems but a passive tube, through which the food passes pretty much as it entered the mouth, giving but little nourishment to the patient and much annoyance to the atonized viscera. In these cases pepsine is very clearly indicated, and will slowly, but quite certainly, aid in the digestion of judiciously selected nutriment, until the system can recuperate sufficiently to manufacture its own pepsine, when the artificial substitute can be withdrawn.

The annexed cases are selected from several given in illustration:

Case 4. July 1st. —— Brush, 18 months old. Severe Diarrhœa.

℞ Pulv. Opii gr. i. Bismuthi Sub. nit. ʒ i. M. et pulv. f. No. xii; S one every three hours.

July 2d. No better. Continue previous prescription.

July 3d. No better.

℞ Am. Pulv. Pepsine ʒ ss. Bismuthi Sub. nit. ʒ i. Pulv. Opii gr. ss. M. et pulv. ft. No. xiii; S one with food every three hours.

July 5th. Much better, and in two days well. Mother informed me three months later that the baby had had several attacks of diarrhœa since, but "a few of those last powders cured him without our needing to send for you."

Case 5. Called July 2d, 9 A. M. ——— Creighton, age 2 years. She has been vomiting and purging severely all night.

℞ Am. Pulv. Pepsine. Bismuthi Sub. nit. aa ʒ i. M. et pulv. ft. No. viii.; S one every three hours, with beef tea.

3 P. M. Vomiting ceased, but much pain and diarrhœa. Added 1 gr. of ipecac, $\frac{1}{8}$ gr. of opium to each powder, and ordered half one to be given every hour.

9 P. M. Better, but pain and frequent passages. Ordered 1 drop Tinct. Opii twice in night, and one-half powder every three hours.

July 3d, 9 A. M. Better, only two passages in 16 hours. Continue the first powder and give 1 drop of Tinct. Opii after each evacuation.

July 4th. Quite smart, but diarrhœa still keeps up a little. Renewed the first prescription; had no need for any other medicine. Perfectly well in two days.

### THE YOUNG DOCTOR.

We clip the following characteristic bit of description from Oliver Wendell Holmes' story, "The Poet at the Breakfast Table," in the *Atlantic Monthly*, March, 1872:

The young Doctor has a very small office and a very large sign, with a transparency at night big enough for an oyster-shop. These young doctors are particularly strong, as I understand, on what they call *diagnosis*—an excellent branch of the healing art, full of satisfaction to the curious practitioner, who likes to give the right Latin name to one's complaint; not quite so satisfactory to the patient, as it is not so very much pleasanter to be bitten by a dog with a collar round his neck telling you that he is called *Snap* or *Teaser*, than by a dog without a collar. Sometimes, in fact, one would a little rather not know the exact name of his complaint, as if he does he is pretty sure to look it out in a medical dictionary, and then if he reads, *This terrible disease is attended with vast suffering and is inevitably mortal*, or any such statement, it is apt to affect him unpleasantly.

I confess to a little shakiness when I knocked at Dr. Benjamin's office door. "Come in!" exclaimed Dr. B. F. in tones that sounded ominous and sepulchral. And I went in.

I don't believe the chambers of the Inquisition ever presented a more alarming array of implements for extracting a confession, than our young Doctor's office did of instruments to make nature tell what was the matter with a poor body.

There were Ophthalmoscopes and Rhinoscopes and Otoscopes and Laryngoscopes and Stethoscopes; and Thermometers and Spirometers and Dynamometers and Sphygmometers and Pleximeters; and Probes and Probangs and all sorts of frightful inquisitive exploring contrivances; and scales to weigh you in, and tests and balances and pumps and electro-magnets and magneto-electric machines ; in short, apparatus for doing everything but turn you inside out.

Dr. Benjamin set me down before his one window and began looking at me with such a superhuman air of sagacity, that I felt like one of those open-breasted clocks which make no secret of their inside arrangements, and almost thought he could see through me as one sees through a shrimp or jelly-fish. First he looked at the place inculpated, which had a sort of greenish-brown color, with his naked eyes, with much corrugation of forehead and fearful concentration of attention ; then through a pocket-glass which he carried. Then he drew back a space, for a perspective view. Then he made me put out my tongue and laid a slip of blue paper on it, which turned red and scared me a little. Next he took my wrist; but instead of counting my pulse in the old-fashioned way, he fastened a machine to it that marked all the beats on a sheet of paper—for all the world like a scale of the heights of mountains, say from Mount Tom up to Chimborazo and then down again, and up again, and so on. In the mean time he asked me all sorts of questions about myself and all my relatives, whether we had been subject to this and that malady, until I felt as if we must some of us have had more or less of them and could not feel quite sure whether Elephantiasis and Beriberi and Progressive Locomotor Atxay did not run in the family.

Therapeutic Action of the Various Modifications of the Galvanic Current.—An article by Dr. Leon Le Fort has appeared in recent numbers of the *Gazette Hebdomadaire*, recommending the substitution of weak continuous currents for continued strong or temporary currents in the treatment of paralysis, muscular contractions and failures of nutrition; and gives the following summary of the relative values of the various modifications of the galvanic current as a therapeutic agent, as at present understood:

"The induced currents, such as are obtained with the apparatus of Duchenne (of Boulogne) and all the other apparatuses used in medicine, appear to be above all useful in exciting artificial movements in muscles over which the will has no control. They prevent simple atrophy, and can also prevent fatty atrophy; placing the muscles in a state of power to obey, later, the commands of the will whenever regeneration shall have taken place in the nervous elements which have become altered or destroyed. It is by this kind of fibrillar gymnastics that they influence the muscular nutrition.

"Perhaps, also, by the excitation which they produce, they can arouse nervous action.

"But there are cases in which these contractions cannot be awakened by the induced currents while continuous currents produce them. The anatomical and physiological conditions of these phenomena are still veiled in much obscurity, but, in brief, it is understood that these currents have only a mechanical action (muscular gymnastics), being useful in the same manner as induced currents, and should be employed in their stead, whenever induced currents are without action. In order to obtain these contractions, one is obliged to make use of currents of strong tension produced by batteries of numbers of elements, such as those employed by Remak, MM. Onimus and Legros.

"Continuous currents which are powerful have a marked action on nutrition; an action which appears to be direct and which shows itself in arresting fatty degeneration by the more rapid reproduction of anatomical elements, and by accelerating the absorption and decomposition of tissues.

"The sedative action of continuous currents, so useful in cases of neuralgia, is even more remarkable in cases of muscular contractions. Induced currents, on the other hand, are more harmful than useful. Now in cases in which it is desired to act upon nutrition—that is to say, upon continuous phenomena, incessant but slow—it appears to me that it would perhaps be advantageous to employ feeble but permanent currents. Experience has justified my predictions as regards paralysis and contractions—will it also be as happy in relation to purely nutritive phenomena? I think not. I have undertaken trials of it in cases of opacities of the crystalline lens, atrophy of the ocular membranes, and it is evident that in these cases one cannot employ strong currents.

"Scientifically, although it may be premature to establish precise indications in a subject still under study, I think I can say that in incomplete paralysis, whenever the induced current can produce muscular contractions, it is necessary to employ induced or interrupted currents.

"In incomplete paralysis, above all if it is the commencement of atrophy, and in complete paralysis in which the current only awakens contractions, it is necessary to employ continuous currents and to use a number of cells whenever it is wished to produce immediate contractions.

"But in all cases of paralysis with simple or fatty atrophy, above all, cases of atrophic reflex paralysis, or in those which are consequent upon a contusion; in all cases when it is desired to favor nutrition of the muscle, it is necessary to employ feeble currents which are permanent or continued for a long time.

"Finally, in all cases of contraction, or paralysis with contraction, feeble and permanent currents should be employed."

The simple apparatus recommended by Dr. Le Fort for the production of a feeble continuous current, and known as the Trouve battery, consists of a common drinking glass, at the bottom of which lies the end of a spiral of copper wire; and a wire of zinc twisted into a knot at its two extremities. One serving for the element, the other to make connection with the adjoining cell. The central wire of copper is isolated from the zinc, and from the column of liquid by a tube of rubber, or of glass, or simply by an oil-varnish. This wire supports the washer of copper which receives the oxide of zinc and prevents its being deposited at the bottom of the vessel on the copper. The glass is to be filled with ordinary water to which have been added several crystals of sulphate of copper, and the apparatus is in condition to work.—*Medical Record.*

On the Treatment of Ingrowing Toe-Nail.—Forty years since, when I was an assistant, a young farmer one day came to the surgery, and was operated upon for an ingrowing toe-nail. This was done by tearing the nail away. The poor fellow suffered so severely that I was impelled to say, "I will never perform that operation." Of course, in many years' active practice, I have had many such cases under my care; and my invariable mode of proceeding has been to find the edge of the nail with a probe, and then remove the whole of the granulations and hypertrophied cellular tissue on both sides, if requisite. In no case have I been disappointed, or ever had to treat the patient for a return of this grievous complaint. I fully expected that this mode of treatment was general; but my attention has been recently drawn to the case of a fine young man rendered almost a cripple by having both his great toe-nails torn out, leaving the overlapping skin to such an extent as to prevent healing, with the probability of return on the growing of a new nail. This was performed by a surgeon to one of the large metropolitan hospitals, where, I am informed, this mode of treatment is taught. In a clinical lecture by another hospital surgeon, reported a few weeks since in the *Journal*, are the following remarks:

"The common mistake made in this case was to blame the nail; but it was not the nail that was at fault, but the skin surrounding. This became thickened and ulcerated, and grew until the nail was overlapped."

Now the mode of operating which I wish to impress is to remove the offending granulations, instead of the unscientific cruel operation of tearing out the nail. I am aware that other surgeons follow this mode of treatment; and I considered it general until my attention was recently drawn to the case above mentioned.—*Stilwell.*—*British Medical Journal, July 13th*, 1872.—*The Clinic.*

Jewish Mortality.—According to Dr. Stallard's work on "London Pauperism," the mortality of Jewish children under five years of age is much less than among other children. There is no hereditary syphilis, and scarcely any scrofula. The mother engages in no employment that takes her away from her children. From one year to five the Jews lose only ten per cent., while the Christians lose fourteen. The average life of the Christian is thirty-seven years—of the Jew, forty-nine years. Beyond sixty years, only a quarter of a Christian population will be alive, but a quarter of a Jewish population will exceed seventy-one years. In Prussia the Christian population requires fifty-one years to double itself, but the Jewish population will double itself in forty-one years.—*Medical Record.*

Transfusion.—An account is given in the *Lancet* of three cases in which transfusion of blood was performed; two successful and one unsuccessful. In the latter, the defibrinated blood had been drawn the night before and kept fourteen hours before being used. This does not seem to have had any influence on the result of the case, which was one of exhaustion produced by gastric ulcer. The cases which were successful were those of poisoning by phosphorus and by carbonic acid. In these a quantity of blood was withdrawn, equal to that injected.—*Ibid.*

"Isolating Dressing of Wounds" is the term used by M. Viennois, in a late number of the *Gazette Hebdomadaire*, to indicate a mode of dressing wounds practiced by M. Olliver for a couple of years past, which he believes to be superior in its effects to the water bath as employed by Langenbeck. In cases where it is possible to do so, the part is immersed in a bath of oil, either by laying it into a trough of zinc, or, in the case of amputation, enclosing the stump in a pig's bladder (in the case of larger limbs) which contains a sufficient quantity of oil to completely cover the wound. The oil, he thinks, being of less s. g. than the fluids of the wound, allows them to gravitate to the bottom of the containing vessel, where they are completely separated from contact with the wounded surface. When this process is not applicable, lint or charpie soaked in oil can be applied, and covered with cotton wadding and a bandage.

Fifty(?) per cent. of phenic alcohol can be added, if desired, to the oil, which may be so rendered antiseptic. Union, in small wounds, sometimes takes place by first intention.—*Medical Record.*

Intestinal Erectile Tumors. — Dr. Laboulbene remarks that erectile tumors have been observed on nearly all parts of the skin, on the orifices of mucous membranes, and in several viscera; but no case of erectile tumor of the stomach or intestine has ever, so far as he knows, been recorded.

He has put the existence of such tumors beyond doubt by a recent communication to the Paris Academy. In the case related by him, the seat of the lesion had been diagnosed by the symptoms, and the forecast was corroborated by the subsequent examination, which discovered a spontaneously-ulcerated tumor of the duodenum.

The author draws the following conclusions:—

1. Erectile tumors (angioma) exist in the intestinal canal as well as the external tegumentary surface.
2. Such tumors are developed on the intestinal mucous membrane.
3. They may give rise to fatal hemorrhage.

Tannate and Sulphate of Quinine. — The question of the activity of the former salt has recently been discussed in the Academy of Medicine of Paris, Messrs. Chanfard, Briquet, and others thinking that it is without activity; and M. Mialhe, speaking of the sulphate of quinine, makes the following observation, which is worthy of attention:—

Sulphate of quinine should never be employed as a basic sulphate, but as an acid salt. In this state the sulphate may compete with all other salts the base of which is quinine; for it should be noticed that it is the quinine itself, and not its saline combinations, which is active. The acid serves only as a vehicle for the introduction of the quinine into the blood, where it is set free by means of the alkaline, or earthy bicarbonates which the blood contains, and then its therapeutic action begins. It is a mistake to believe that the sulphate of quinine, when the salt is administered, can be traced in the urine; the salts traced are the acid phosphates of quinine associated with the phosphates of lime and magnesia.—*Ibid.*

Renal Calculi the Cause of Screaming-Fits in Infants.—At a late meeting of the New York Pathological Society, Dr. Jacobi said that the frequency with which stones were found in the kidneys of new-born infants would lead to the conclusion that the origin of calculi was, if not congenital, confined to the earlier weeks of infancy. In forty *post mortems* upon children under six months, made in succession, there were calculi in the kidney in six. He remarked that the violent screaming-fits met with in young infants could often be accounted for on the supposition of the passage of a renal calculus. He had met with two such cases; one he had known to have had several of such screaming-fits, and in the other the stone was cut out and was found to have a very large nucleus of uric acid, pointing most unequivocally to its renal origin.—*Medical Press and Circular.*

American Children. — The foreign element in this country breeds more than double as many children as the strictly American. In the New England States, formerly, every family averaged about eight children. From the best information of the present day, it seems that in the families of the native population in those States they do not exceed three.—*Medical Record.*

A Collection of Pelves.—The Obstetrical Society of London has appointed a committee to form a collection of pelves, illustrative of various points in connection with obstetrics. The Honorable Secretaries to the Pelvis Committee, Alfred Wiltshire, M. D., and Heywood Smith, M. B., state that they will be willing, if desired, to make arrangements as to expense of carriage, casts, models, drawings, photographs, etc., with any society in this country, who may wish to contribute specimens. Their address is at the Society's Library, 29 Regent street, W.—*Ibid.*

Action of Phosphorus on the Bones.—At the Surgical Congress, held at Berlin in April, Herr George Wagner detailed the results of his experiments on the action of phosphorus on the organism, so far as they are of surgical interest. He finds that the fumes of phosphorus always produce periostitis of the jaws and facial bones in animals when the periosteum has been injured; when this membrane has not been injured, this effect is only occasionally observed. Phosphorus vapor also acts as an irritant on the exposed periosteum of other bones, but less intensely than on the jaws. Phosphorus given internally in pills, or in the form of phosphorus acid, affects the bones generally; but the results vary according as the animal is growing or full-grown. In growing animals new bone is deposited in much thicker masses than in the normal state. For instance, at the end of the diaphyses of the joints and at the beginning of the epiphyses of the long bones, the cancellated tissue is replaced by perfectly compact bone; and those parts of the bone which are formed from the periosteum undergo thickening, to such an extent as even to obliterate the Haversian canals. In grown animals the cancelli and the Haversian canals are narrowed; and in the tubular bones the medullary canal is encroached on by an osseous layer, and has even been observed, in fowls, to become completely obliterated. When the bones have been artificially injured by fracture or resection, the effect of the internal administration of phosphorus is to produce a richer and thicker deposit of new bone, presenting, especially after fracture, the character of ivory. The effect on osteoplasis is produced by the daily use of doses so small as not produce any toxic results, even though long continued. The practical question raised by Herr Wagner was, whether these results did not point out that phosphorus might be found useful in osteomalacia and in fractures, and also in caries and rickets.—*Brit. Med. Journ.*, June 15, 1872.

Deaths from Chloroform.—On June 8, an inquest was held at King's College Hospital, on the body of William Lyon, aged 36, who was an engine-driver, and had been under treatment at the hospital for an affection of the jaw-bone. It was considered necessary to perform an operation, and he was placed under the influence of chloroform, and two minutes after its administration he expired. A verdict that deceased died from paralysis of the heart, caused by chloroform, was returned.

The death of a patient while on the operating-table, under the influence of chloroform, occurred on the 27th of last month in the practice of the eminent Vienna surgeon Billroth. Disarticulation at the hip-joint was being performed, on account of osteomyelitis. The femoral artery had been tied, and Billroth was about to apply the galvano-caustic wire in order to divide the soft parts after the enucleation of the head of the bone, when the patient's breathing became stertorous, and he died. Tracheotomy was immediately performed; but it and all other attempts to restore life were fruitless.—*Brit. Med. Journ.*, June 15, 1872.

Vital Statistics of France and Prussia.—A considerable sensation has been caused this week in France by the communication of M. Decaisne to the Academy of Medicine of an important study on the depopulation of France, indicated by its present vital statistics. Comparing it with Prussia, he shows that there 100 marriages produce 400 children, while in France they give only 300. The percentage of births to population in Prussia is 3.98; in France, only 2.55. The annual excess of births over deaths in Prussia is 13,000 per million; in France, 2,400. As to the future, the French population would require one hundred and seventy years to double itself; that of Prussia, only forty-two; that of Great Britain, fifty-two; that of Russia, sixty years.—*Brit. Med. Journ.*, June 15, 1872.

Amyloid Degeneration.—It is well known that amyloid degeneration is very common after long-continued suppurations, especially of bones and joints. But Prof. Cohnheim gives (*Virchow's Archiv*, vol. liv., pts. i. and ii.) three cases in which it was found, after a suppuration of joints of only some months' duration. The suppurations occurred in healthy men from wounds received in battle. The spleen was affected in all the three, and the kidney in one, so that the amyloid degeneration was in an early stage. The author has no doubt that the degeneration was the result of the suppuration, and the cases are remarkable on account of its occurring after such a short period.—*Glasgow Med. Journ.*, May, 1872.

The First to Suggest the Idea of Dispensaries.—Dr. Samuel Garth, in 1702, was the first physician who suggested the idea of dispensaries where advice was given gratis to the poor. He may be considered the founder of those benevolent institutions. His poem of "The Dispensary" was a satire on the interested quacks and apothecaries who opposed the charity.—*Medical Record.*

Nervous or Sick Headache.—In those forms of sickness which are preceded by disturbed vision, or other signs recognizable by the patient as preceding an attack, the patient should lie down with the head as low as possible, and if the glimmering be on the right or left of the field of vision, he should lie on the opposite side. He should at the same time take some powerful diffusable stimulant. By this means the defective supply of blood to some portion of brain, which is the real disease, is counteracted. There is always a loss of tone about the cerebro-spinal system in cases of this kind, and of course all measures calculated to improve the general health should be adopted. If the attack is followed or preceded by great mental depression, nothing acts like half a drachm or a drachm of the ammoniated tincture of valerian. A remedy which is often given with great advantage during a severe attack is bromide of potassium in doses of five, ten, to thirty grains, combined with thirty or forty minims of sal volatile. If the attacks have been very frequent, or if there be any scrofulous tendency, the iodide of iron may be given in the following form:

℞ Ferri et ammon. cit., gr. v.;

Potassii iodidi, gr. ij.;

Aquæ, ℥ j.; and, according to circumstances, fifteen to twenty minims of tincture of henbane, or twenty to thirty minims of aromatic spirit of ammonia may be added. If the stomach is irritable this may be given in the effervescing form. In other cases citrate of iron with ammonia and strychnine may be given with great success.—*Dr. P. W. Latham, p.* 102.—*Medical Press and Circular.*

Neuralgia. — *Galvanism.* — In order to cure neuralgia by galvanism we should use the continuous current, by means of small wet sponges attached to small conical conductors. The constant galvanic current has a truly marvelous effect over pain, whereas the interrupted current is of little or no service. The battery used should be Weiss's, sometimes known as Foveaux's. Eight cells of this splendid battery suffice, with very small sponges (about as large as would fill the head of a thimble). They should be applied to the painful part, an inch or two apart, and moved about, without being actually removed from the skin, for about two minutes. After resting a minute they should then be applied again for two minutes. Three very interesting cases illustrative of this plan of treatment are given.—*Mr. J. Stead, p.* 112.—*Medical Press and Circular.*

